# Nodular Pulmonary Amyloidosis: A Manifestation of Sjögren's Syndrome

**DOI:** 10.1155/2018/9745935

**Published:** 2018-08-13

**Authors:** Gustavo Adolfo Gómez Correa, Jovani Osorno Serna, Manuel Felipe Cáceres Acosta, Juan David Cáceres González, Jaime Andrés Calle Ramirez, Juan Paulo Sandoval Mesa, Miguel Ignacio Roldán Pérez

**Affiliations:** ^1^Specialist in Internal Medicine and Pneumology, Department of Internal Medicine, Universidad de Antioquia, San Vicente Foundation University Hospital, Colombia; ^2^Specialist in Internal Medicine, Pneumology, Thoracic Oncology and Epidemiology, Department of Internal Medicine, Universidad de Antioquia, San Vicente Foundation University Hospital, Colombia; ^3^Resident of Internal Medicine, Universidad del Cauca, Colombia; ^4^Undergraduate Medical Student, Universidad de Antioquia, Colombia; ^5^Resident of Internal Medicine, Universidad de Antioquia, Colombia; ^6^Resident of Pathology, Universidad de Antioquia, Colombia; ^7^Specialist in Pathology, Department of Pathology, Universidad de Antioquia, San Vicente Foundation University Hospital, Colombia

## Abstract

**Case Description:**

A 72-year-old woman with primary Sjögren Syndrome (SS) was diagnosed during an inpatient hospital stay with dry symptoms. The patient had respiratory and constitutional symptoms, requiring a pulmonary imaging evaluation by high-resolution computed tomography (HRCT) of the thorax.

**Clinical Findings:**

Multiple cavitary pulmonary nodules, halo sign, and focal areas of ground-glass opacity with predominance in both bases were found in the images. Complementary studies were done where a neoplastic focus was ruled out. The diagnosis of nodular pulmonary amyloidosis was confirmed with a pulmonary biopsy performed by videothoracoscopy for histopathological study, which reported the formation of nodules in the parenchyma with amyloid deposits and positive immunohistochemical markers for CD3, CD20, and CD38 lymphocytic infiltration.

**Treatment and Outcome:**

Initial inpatient management with intravenous cyclophosphamide and methylprednisolone was given. Subsequent outpatient management was given with high dose glucocorticoids.

**Clinical Relevance:**

We presented a case of nodular pulmonary amyloidosis in a female patient with primary SS, which is a rare pulmonary manifestation of this syndrome.

## 1. Introduction

Amyloidosis is part of a heterogeneous group of disorders associated with the extracellular deposit of abnormal proteins formed by amyloid fibrils in various organs. It can be localized affecting a single organ or systemic affecting multiple organs [1]. The pulmonary presentation may be tracheobronchial, nodular parenchymal [amyloidoma], or diffuse parenchymal [interstitial/diffuse alveoloseptal] [2, 3]. Pulmonary nodules, with calcification and cavitation, are common findings in the thoracic image, being a good prognosis. We describe the first reported case in Colombia of nodular pulmonary amyloidosis associated with Sjögren Syndrome (SS).

## 2. Case Description

The case is of a 72-year-old female, chronic smoker patient with a 3 pack-years until 20 years ago, with a personal history of hypertension, obesity, dyslipidemia, breast nodules, and transverse myelitis with motor sequelae. She has been hospitalized 6 years ago for community-acquired pneumonia.

**Figure 1:**
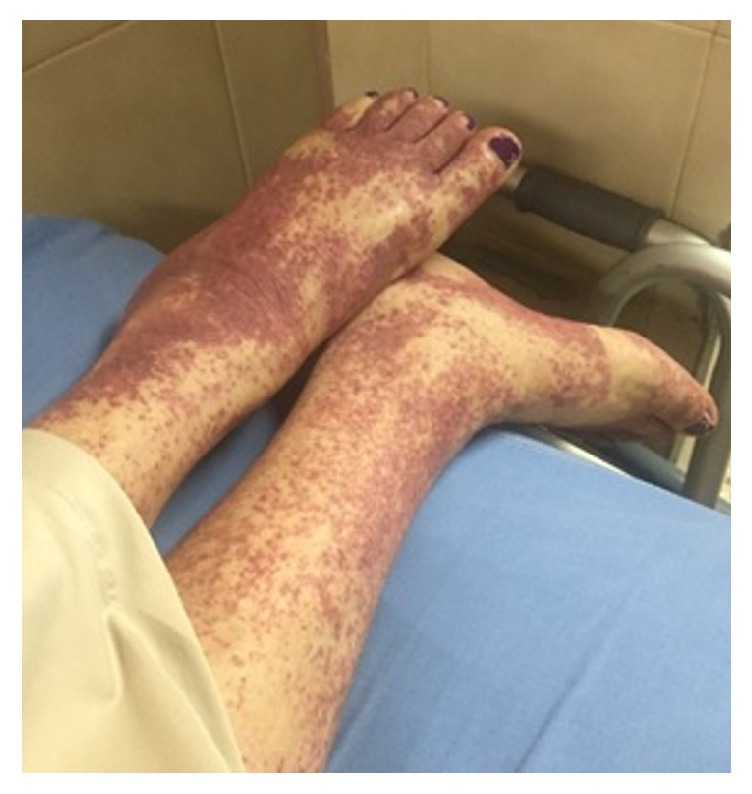
*Nonpalpable purpura on lower limbs*. Multiple dispersed petechiae are observed, which converge in their great majority in the left lower limb.

She consulted for several months of asthenia, adynamia, hyporexia, and subjective weight loss. Associated with this, she had 10 days of fever, malaise, myalgias, non-palpable purpura on the lower limbs (Figure 1), mild dyspnea, and intermittent cough without hemoptysis.

She had dry symptoms (xerostomia and xerophthalmia) initiating a study for primary SS during hospitalization. She had a positive Schirmer tear test, antinuclear antibodies 1:160 with a speckled pattern, positive anti-Ro/SSA, and anti-La/SSB, as well as positive rheumatoid factor and mild C3 hypocomplementemia. The diagnosis of SS was confirmed with a minor salivary gland biopsy that reported chronic sialadenitis with Chisholm-Mason grade of 4.

Her physical examination did not present hemodynamic instability; she was afebrile with a few bibasilar rhonchi without respiratory difficulty. She had urinary incontinence, nonpalpable purpuric lesions on the lower limbs, and decreased distal muscular strength, partially limiting the gait. During her stay, she presented respiratory and metabolic acidosis with a blood urea nitrogen/creatinine ratio> 20 that was corrected with supplemental oxygen and intravenous fluids. Her exams were performed, including a complete blood count, complete liver function tests, serum electrolytes, and acute phase reactants, which were found in normal ranges. She also had negative hepatitis C virus (HCV) antibody test and nonreactive tests for human immunodeficiency virus (HIV) and syphilis. The serum protein electrophoresis: mild broad-based peak in the gamma region (<3 g/dL) and skin biopsy reported capillaritis with no histological signs of malignancy.

The possible associated pathologies in SS, like non-Hodgkin lymphoma, mainly of mucosa-associated lymphoid tissue (MALT) type, ANCA-associated vasculitis, and cryoglobulinemic vasculitis were ruled out.

**Figure 2:**
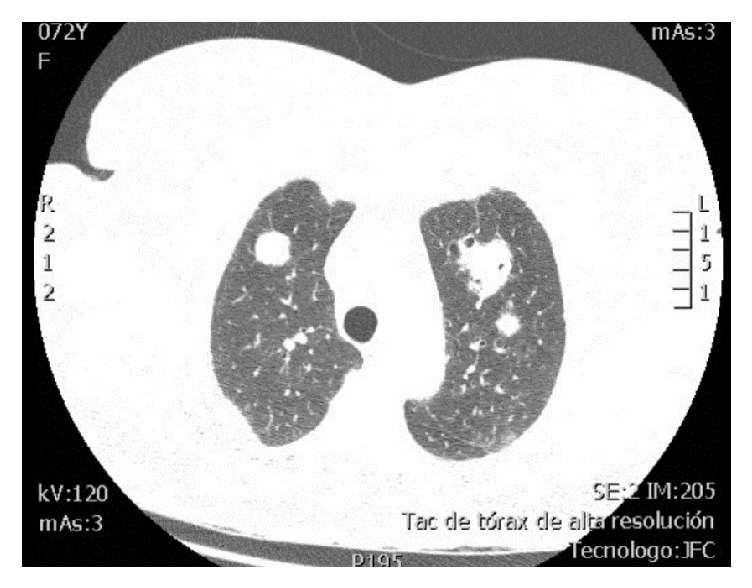
*HRCT of the thorax, axial view, pulmonary window*. There are multiple nodules with random distribution in both lung fields, some of which demonstrate discrete halo sign in ground-glass opacity and others demonstrate cavitation. The largest size is subpleural, in the posterior segment of the right upper lobe, measuring 23 x 32 x 30 mm.

**Figure 3:**
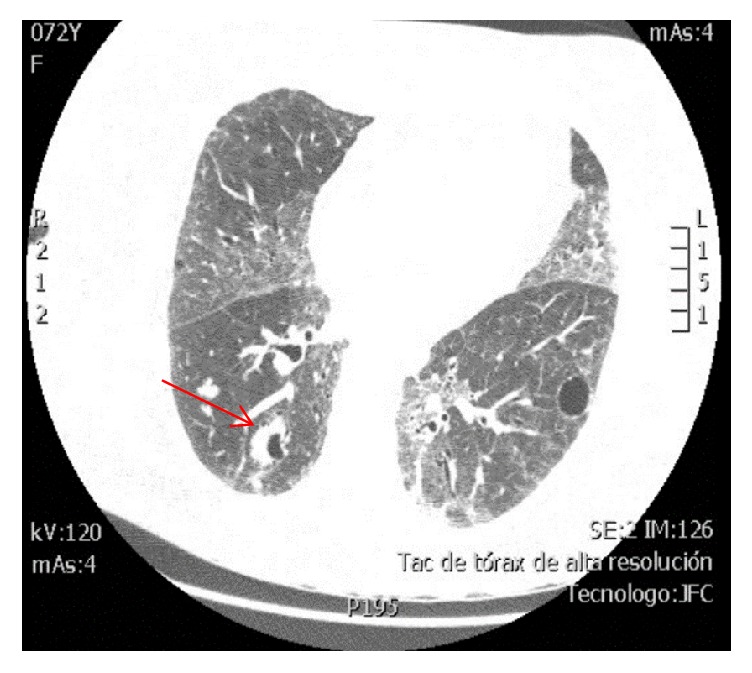
*HRCT of the thorax, axial view, pulmonary window*. Right lung with cavitated nodules, noncalcified, with the halo sign evident in one of them (red arrow). Areas of ground-glass opacity in the middle lobe, lingula, and inferior lobes, with thickening of intralobular septa. There are some residual cysts.

In this context, due to pulmonary nodular involvement evidenced in the non-contrast computed tomography (CT) scan of the chest, an HRCT was requested (Figures 2 and 3) which showed multiple generalized, noncalcified nodular lesions, some with cavitations, and the presence of focal areas of ground-glass opacity associated with suggestive basal predominance subpleural cysts that are suggestive of lymphocytic pneumonia. The CT-guided lung biopsy was considered to clarify its etiology as there were no findings of neoplastic foci in the extension studies (non-contrast head CT scan, non-contrast abdominal CT scan, transthoracic echocardiography, mammography, and upper digestive endoscopy).

Fiberoptic bronchoscopy did not report any anatomical abnormalities or evident lesions in the pharynx, hypopharynx, larynx, main bronchus, interlobar bronchus, lobular bronchus, or segmental bronchus. In the left main bronchus, there were scarce and diffuse mucohemorrhagic secretions in the bronchial tree, predominating in the bronchus for the upper left lobe, with no evidence of apparent active bleeding. Microorganisms were not isolated in bronchoalveolar lavage. Bronchial brushing showed a benign cytological pattern, acute inflammation, and negative special staining for mycobacteria and fungi.

**Figure 4:**
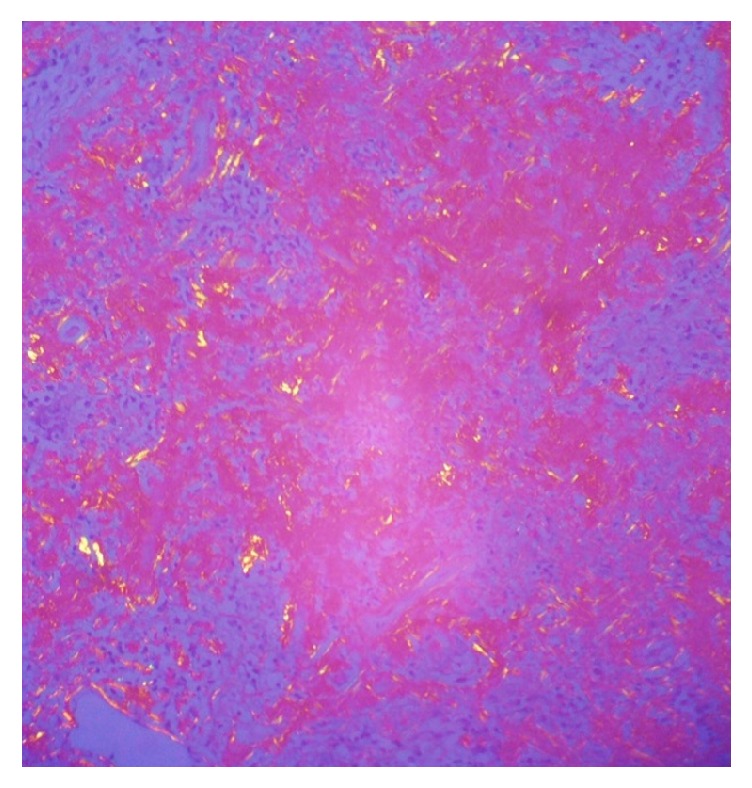
*Hematoxylin and eosin stain in lung biopsy. 4X*. Pulmonary parenchyma with distortion of its architecture, anthracite pigment is observed, and alveolar septa thickened with inflammatory infiltrate of lymphocyte predominance. Presence of peribronchiolar lymphocytic inflammatory infiltrate in some lymphoid nodules.

**Figure 5:**
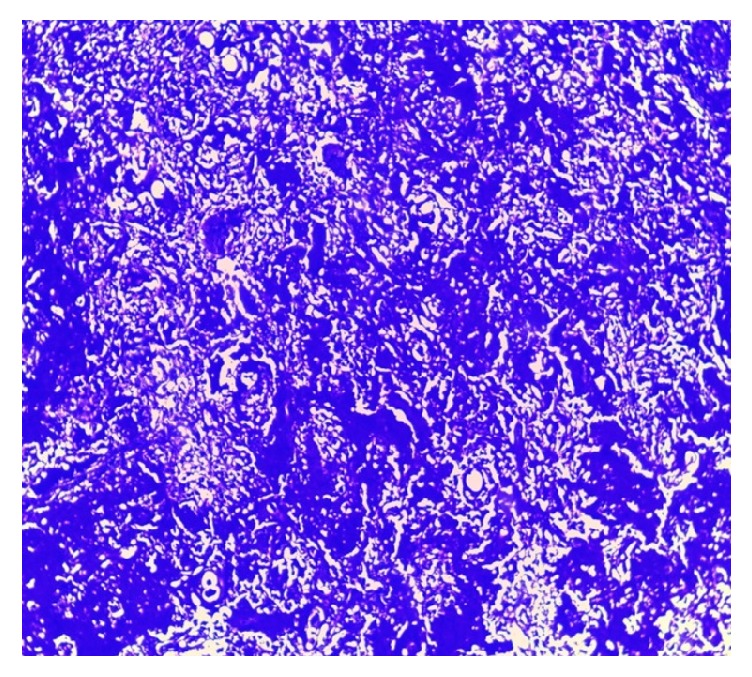
*Crystal violet staining in lung biopsy. 4X*. Pulmonary parenchyma where nodules are made up of dense amorphous eosinophilic material that captures the coloration, which confirms the presence of amyloid. In the middle of it, there are inflammatory cells of the lymphoplasmacytic type.

**Figure 6:**
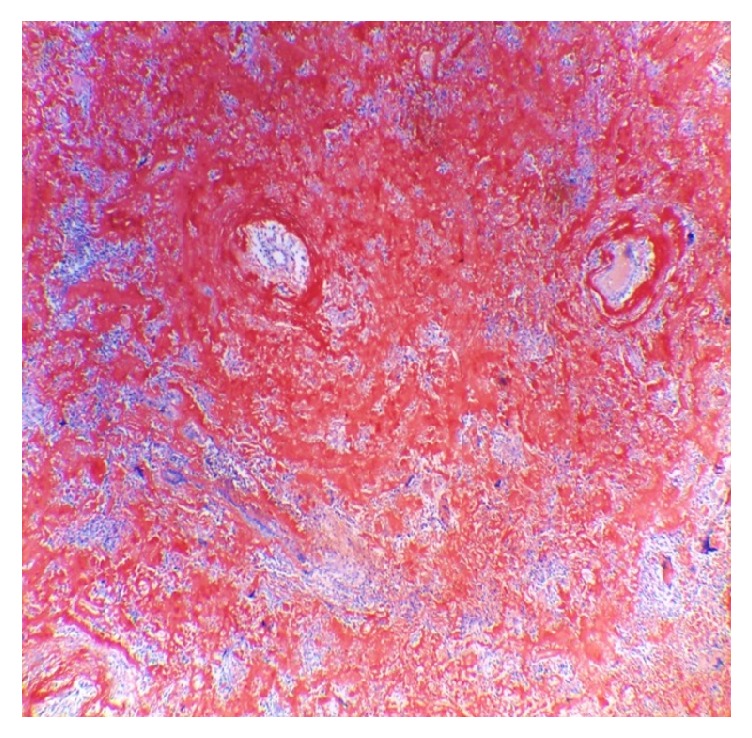
*Congo red staining in lung biopsy. 4X*. Pulmonary parenchyma with distortion of its architecture, thickened alveolar septa, and nodules constituted by dense amorphous eosinophilic material that captures the coloration, accompanied by lymphoplasmacytic inflammatory cells.

Subsequently, video-assisted thoracoscopy (VATS) was performed with pulmonary biopsy. The biopsy of the upper lobe and lingula of the left lung had as findings distortion of the parenchyma architecture, pleural thickening with the presence of anthracite pigment, and alveolar septa thickened with inflammatory infiltration of lymphocyte predominance. Presence of inflammatory infiltration of peribronchiolar lymphocyte predominance in some lymphoid nodules is shown in Figure 4. There was formation of nodules with dense amorphous eosinophilic material, in the middle of which there are lymphoplasmacytic inflammatory cells. In addition, with positive CD3: immunohistochemical markers for reactive T lymphocytes, CD20: for reactive B lymphocytes, and CD38: for plasma cells and kappa and lambda positivity, similar areas of nodular formation were found with amyloid deposition using special staining for crystal violet (Figure 5) and Congo red (Figure 6).

With these findings, the diagnosis of nodular pulmonary amyloidosis and non-specific interstitial pneumonia (NSIP) was made. Both pathologies were explained by the SS. Inpatient immunosuppressive treatment was with cyclophosphamide 800 mg IV single dose and methylprednisolone 150 mg IV single dose; subsequently, on an outpatient basis, prednisolone 50 mg PO qd was administered for one month with gradual reduction.

## 3. Discussion

In the previous case report, we show how step by step the final diagnosis of the patient was achieved, taking into account a rheumatic disease such as SS. This chronic inflammatory disorder is recognized as a lymphoproliferative disease with variable presentation since polyclonal lymphocytic infiltration of the salivary glands until the proliferation of oligoclonal or monoclonal B cells that can generate clonal disorders such as monoclonal gammopathy, light-chain amyloidosis (AL), malignant lymphoma, and mucosa-associated lymphoid tissue (MALT) lymphoma [4, 5]. Pulmonary involvement of the primary SS can occur with parenchymal, pleural, respiratory, or vascular manifestations [6, 7]. The amyloid deposits in patients with SS are even more uncommon; it can affect multiple organs, including the lung [8, 9]. The first description of amyloidosis located in the lower respiratory tract was made by Lesser in 1877 [10]. Pulmonary involvement is incidental because its asymptomatic course is common. The age of presentation is around 65 years and there are no differences with respect to gender [11]. It can be found in tomographic images with cystic or nodular forms mainly in the lower lobes. The most common findings in the HRCT are lobulated contours, calcifications, different sizes 0.5-15 cm, and slowly growing without regression of lesions; cavitation is very rare [12, 13]. In lung biopsy, amyloid deposits were identified, which show green birefringence when stained with Congo red and are visible under polarized light [14]. In the case, we found cystic and nodular lesions at the lung level along with systemic symptoms and pulmonary biopsy by VATS that guided the diagnosis of nodular pulmonary amyloidosis.

## 4. Conclusions

Nodular pulmonary amyloidosis is a rare disease, which should always be considered in patients with multiple lung nodules and its concomitance with SS and MALT. Always rule out granulomatous diseases, primary neoplasm, or metastasis. Corticosteroids therapy is the basis of the treatment, having a good prognosis.
